# Editorial: Nanomaterials and multimodal tumor therapy

**DOI:** 10.3389/fonc.2022.1081687

**Published:** 2022-12-08

**Authors:** Yongqi Yang, Qiaohui Chen, Yige Qiu, Yiying Wang, Qiong Huang, Kelong Ai

**Affiliations:** ^1^ Xiangya School of Pharmaceutical Sciences, Central South University, Changsha, Hunan, China; ^2^ Hunan Provincial Key Laboratory of Cardiovascular Research, Xiangya School of Pharmaceutical Sciences, Central South University, Changsha, China; ^3^ Department of Pharmacy, Xiangya Hospital, Central South University, Changsha, Hunan, China; ^4^ National Clinical Research Center for Geriatric Disorders, Xiangya Hospital, Central South University, Changsha, Hunan, China

**Keywords:** nanomaterials, multimodal therapy, drug delivery, cancer treatment, tumor microenvironment

Although cancer treatment has improved dramatically over recent decades, it continues to remain among the most challenging public health issues. Comprehensive cancer management continues to evolve from use of earlier modalities such as surgery, radiotherapy (RT) and chemotherapy (CHT), to increasingly sophisticated approaches incorporating thermal therapy (e.g., photothermal therapy (PTT), magnetic-thermal therapy), dynamic therapy (e.g., photodynamic therapy (PDT), chemodynamic therapy (CDT), sonodynamic therapy (SDT) (1)) and more recently, immunotherapy (2). Such emerging therapies minimize tissue trauma, enhance targeting of malignant cells, reduce systemic side effects and improve patient prognosis.

However, monomodal therapeutic strategies that depend on a single mechanism of action are often inherently flawed. For instance, efficacy of thermal ablation by PTT is often lessened by expression of heat shock proteins produced under stress; treatment of deep-tissue tumors is thus often limited by the depth of light penetration. Oxygen-dependent dynamic therapy, which utilizes oxygen to produce reactive oxygen species (ROS) particularly toxic to cancer cells, is known to exacerbate hypoxia in the tumor microenvironment (TME) and thus exhibit poor efficacy. Similarly, tumor-driven immunosuppression is known to frequently render immunotherapy (IT) ineffective. Development of multimodal tumor therapies is thus urgently warranted.

Major advances in nanotechnology have paved the way for the exploitation of multi-functional therapeutic platforms. Elaborate nanoplatforms constructed *via* drug loading, structure optimization and surface modification, can thus be expected to exhibit efficacious anti-tumor activity.

In comparison with monomodal tumor therapy, nanomaterial-based multimodal therapy (**Figure 1**) possesses major advantages. First, use of multimodal therapy can exert multi-faceted anti-neoplastic effects while minimizing risks of multidrug resistance and untargeted toxicity. Second, multimodal therapeutic strategies can apply precision medicine approaches to treat a variety of malignancies. Third, nanomaterial optimization can effectively achieve controlled drug delivery to tumor tissues and thus significantly improve pharmacotherapeutic outcomes.

**Figure 1:**
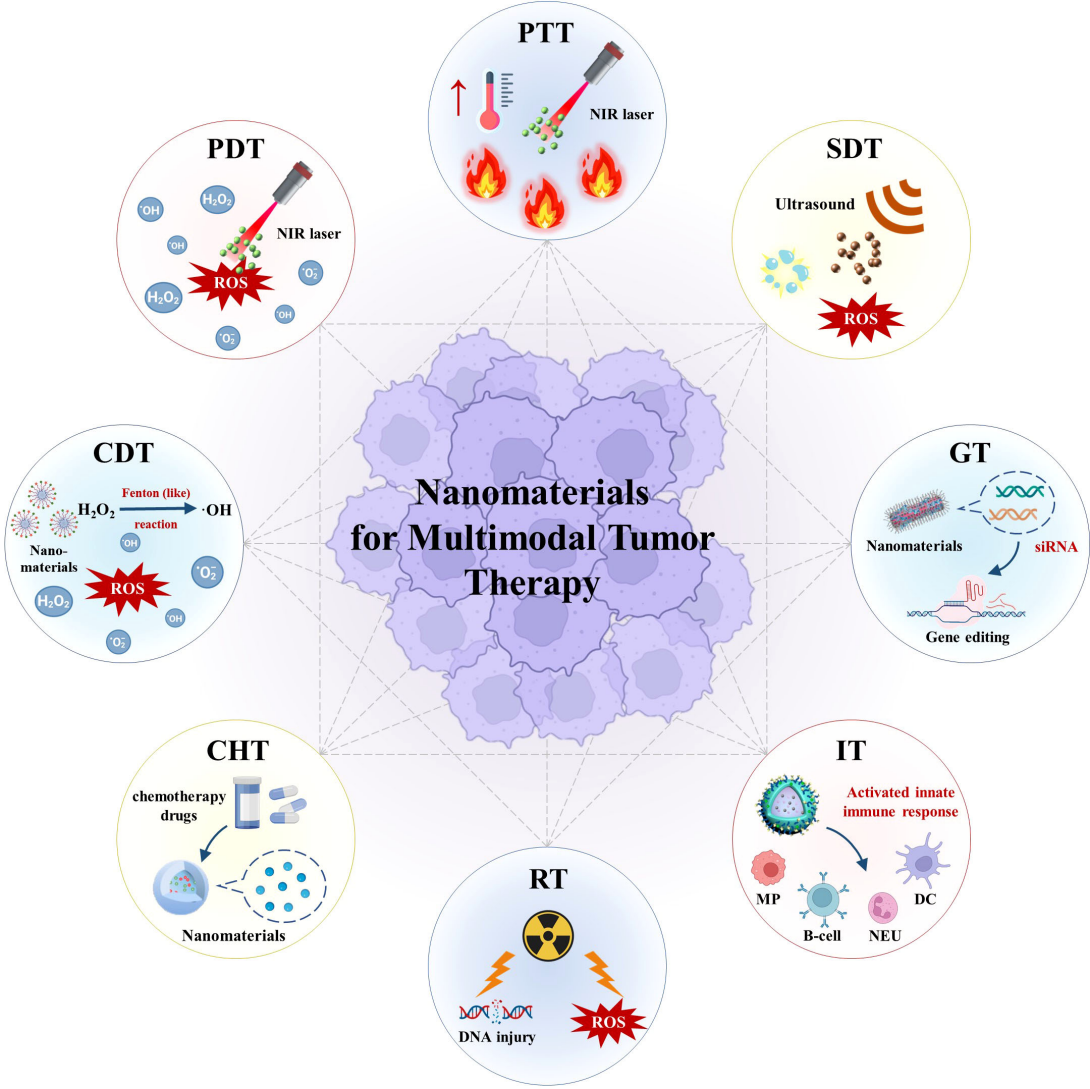
Schematic illustration of nanomaterials for multimodal tumor therapy. (PTT, photothermal therapy; PDT, photodynamic therapy; NIR, near-infrared; ROS, reactive oxygen species; CDT, chemodynamic therapy; SDT, sonodynamic therapy; CHT, chemotherapy; RT, radiotherapy; GT, gene therapy; IT, immunotherapy; MP, macrophage; NEU, neutrophil; DC , dendritic cell).

As mentioned by Shi et al., combination strategies based on synergistically enhanced interactions among two or more treatments tend to produce super-additive “1+1>2” effects. They introduced common inorganic, organic and composite photothermal nanomaterials in the context of multimodal cancer treatment, highlighting the great potential of photothermal nanomaterial use.

In PTT/PDT dual-modal therapy, PTT achieves thermal ablation *via* near-infrared (NIR) light irradiation utilizing a specific wavelength, while PDT kills cells *via* photosensitization and induction of cytotoxic ROS generation. Zhu et al. prepared NIR-responsive injectable agarose hydrogels containing Cu-Hemin and indocyanine green (ICG). As a photothermal agent, ICG was found to effectually absorb and convert NIR light energy emitted by an 808 nm laser into thermal energy. Resultant heating and softening of the hydrogel matrix released Cu-Hemin to mediate antioxidant glutathione (GSH) consumption. Furthermore, ICG was reported to act as a photosensitizer that facilitated production of significant quantities of cytotoxic ROS in tumor cells. As such, PTT/PDT therapy utilizing Cu-Hemin and ICG effectively modulates GSH content within the TME and amplifies the anti-neoplastic effects of PDT.

Utilizing low-intensity ultrasound to generate ROS within tumor cells, SDT has been shown to effectively induce apoptosis. However, marked oxygen consumption in the setting of SDT was reported to aggravate TME hypoxia and thus limit its therapeutic use. Wang et al. designed a multifunctional hydrogel capable of co-delivering nanozyme prussian blue (PB) and sonosensitizer chlorin e6 (Ce6). After photoirradiation using an 808 nm laser, PB was found to convert light energy into heat. After heat-induced hydrogel softening, release of PB and Ce6 was achieved. Resultant catalysis of endogenous H_2_O_2_ to O_2_ by PB alleviated hypoxia within the TME and enhanced Ce6-mediated SDT. Both *in vivo* and *in vitro* results have underscored promise in utilizing PTT/SDT synergy in cancer treatment.

Autophagy was reported to render pharmacotherapy ineffective by facilitating drug resistance among cancer cells, which assists to clear damaged and aging organelles timely and maintains their persistent abnormal proliferation. As such, clinical use of PTT-induced thermal ablation remains challenging. Zhang et al. designed a PTT/gene therapy (GT) platform to load transcription factor EB (TFEB)-siRNA by cell penetrating membrane peptides (CPP)-functionalized gold nanorods (GNRs) with good stable photothermal conversion rate. Importantly, use of CPP was found to enhance the cell permeability of GNRs. Under NIR irradiation, this platform accurately delivered siRNA targeting the autolysosomes, which could weaken the drug resistance of osteosarcoma cells and enhance PTT efficacy. In addition, TFEB silencing by this platform was reported to efficiently inhibit pulmonary metastasis by osteosarcoma cells because TFEB associated autophagy with lysosomal biogenesis and promoted cancer metastasis.

Although generation of hydroxyl radicals (·OH) *via* Fenton or Fenton-like reactions triggers oxidative damage, CDT efficiency remains limited by the delivery rate of Fenton reagents and complex TME conditions. Qin et al. attempted to synergistically enhance the Fenton reaction utilizing PTT/CDT by developing a novel NIR-mediated, tumor-specific nanoplatform based on magnetic iron oxide nanoclusters (MNCs). MNCs were found to possess excellent magnetic response characteristics and facilitate delivery of Fenton reagents to tumor sites *via* magnetophoresis. Furthermore, MNCs exhibited excellent photothermal characteristics and Fenton-like nano-catalysts activity, which accelerated Fenton reactivity and enhanced therapeutic effects *via* an increase in temperature. As such, incorporation of PTT and the Fenton reaction was found to produce superior anti-tumor effects as compared to monotherapy both *in vitro* and *in vivo*, with MNCs + NIR group almost completely inhibiting *in vivo* tumor growth. Similarly, Zeng et al. developed a chemodynamic hydrogel system based on iron-gallic acid nanoparticles (FeGA) and α-cyano-4-hydroxycinnamic acid (α-CHCA) that also utilized PTT/CDT co-therapy. Irradiation of FeGA by an 808 nm NIR laser resulted in conversion of light energy into thermal energy, with Fe^2+^ simultaneously enhancing Fenton reactivity to damage tumor cells. Furthermore, diffusion of α-CHCA into the TME was found to increase intracellular acidity and contribute to ·OH production by CDT.

Profound nonspecific side effects of CHT significantly affect patient quality of life and limit patient compliance to treatment. As such, nanomaterials-based combination therapy utilizing PTT and CHT has recently emerged as a focus of study. Zhao et al. designed a biomimetic nanosystem based on a zeolitic imidazolate framework 8 (ZIF8) that employed particles loaded with doxorubicin hydrochloride (DOX·HCl) and photothermal agent copper sulfide (CuS), and that were coated with red cell membrane and catalase (CAT) components. With its biodegradation dependent on pH, ZIF8 rapidly degraded in the acidic TME and facilitated targeted co-delivery of both CHT and PTT agents. Furthermore, catalysis of H_2_O_2_ decomposition functioned to provide sufficient O_2_ for PTT activity, thus enhancing DOX delivery and cytotoxicity. Combined utilization of PTT and RT was similarly reported to optimize treatment while lowering required radiation doses and minimizing side effects. Hydrogel-encapsulated SnFe_2_O_4_ (SFO) nanozyme was reported by Zeng et al. to exploit GSH oxidase (GSH-OXD) and CAT activity in addition to PTT effects, thus reducing GSH levels in tumor tissues and effectively catalyzing formation of large quantities of O_2_. Thus, the anti-cancer properties of RT were enhanced *via* alleviation of hypoxia within the TME and minimization of resistance. *In vivo* anti-tumor findings revealed almost complete inhibition of tumor growth in the PTT/RT group. Moreover, Huang et al. highlighted the advantages of Bi_2_X_3_-based nanomaterials as photothermal agents in the context of PTT enhancement that improves ROS production in RT and thus broadens the clinical application of Bi_2_X_3_-based nanomaterials in RT use.

In addition to PTT-based strategies, other bimodal approaches also optimize multi-faceted therapeutic outcomes. Incorporation of CDT and RT by Zhang et al. entailed fixation of glucose oxidase (GOD) and Hemin on the surface of biodegradable mesoporous silica nanospheres (MSN). Oxidation of glucose by GOD generated significant quantities of H_2_O_2_; subsequent conversion of H_2_O_2_ into ROS by Hemin markedly enhanced RT efficacy and produced an *in-vivo* tumor suppression rate as high as 91.5%. Chen et al. designed a CHT/RT platform employing platelet membrane-coated gold nanocages (AuNs) loaded with cisplatin capable of directly destroying tumor cell DNA and enhancing RT efficacy. This platform almost completely inhibited increases in tumor volume *in vivo*. Yang et al. encapsulated camptothecin (CPT) and the CO prodrug MnCO in ZIF8 nanocarriers that decomposed in the acidic TME, releasing CPT to destroy tumor mass and produce significant quantities of H_2_O_2_. Subsequently, MnCO triggered a Fenton-like reaction with H_2_O_2_ to generate CO; this localized increase in CO levels inhibited mitochondrial respiration and induced tumor cell apoptosis. Such enhancement of CHT in the setting of CDT was reported to suppress approximately 90% of tumor cells *in vitro* and almost entirely inhibit tumor volume growth in mice with CT26 tumors.

Importantly, multimodality was reported to exhibit even greater superiority over monomodal and bimodal approaches. Feng et al. constructed an intelligent PTT/CHT/IT nanoplatform using Mn^III^PC@DTX@poly-acylcosugar acid (PLGA)@Mn^2+^@hyaluronic acid (HA) (MDPMH) for treatment of non-small cell lung cancer. Mn-modified phthalocyanine derivative (Mn^III^PC) was employed as a photothermal agent to facilitate PTT while docetaxel (DTX) was delivered in a targeted fashion to the tumor site to minimize non-targeted toxicity to normal tissues. Endocytosis of Mn^2+^ by macrophages, dendritic cells and lymphocytes resulted in activation of the innate immune response. *In vitro* findings confirmed this platform to be significantly more effective in inducing tumor cell apoptosis as compared to monomodal or bimodal strategies.

Use of multimodal strategies in concert with PTT/CHT/CDT overcomes a number of practical limitations such as penetration depth in PTT and decreases drug resistance. Wang et al. studied use of encapsulated CPT and pyrite (FeS_2_) with injectable hydrogels. A photothermal agent, FeS_2_ simultaneously functioned as a peroxidase (POD), mediated PTT and catalyzed production of ·OH within the TME *via* Fenton reactivity. The GSH-OXD activity of FeS_2_ resulted in consumption of excess intracellular GSH and further amplified oxidative stress. Moreover, this hydrogel system allowed for controlled release of nanomaterials, enhancing the selective chemotherapeutic effect of CPT, maintaining within the therapeutic window, and ultimately showing favorable tumor-specific cytotoxicity. Of note, PTT/CHT/CDT treatment group was reported to be most efficacious in mice.

In summary, incorporation of existing nanomaterial-based drug delivery platforms to rationally design multimodal cancer treatment strategies holds great promise for future clinical applications. Their cooperations are typically complementary between two or more therapies, including, but not limited to, examples within this Research Topic. But it is regrettable that research progress in this area has remained slow and most of the aforementioned therapies remain at the laboratory level. As such, further systematic study of potential contradictions and side effects in the context of multimodal treatment strategies is urgently warranted.

The emerging field of nanomedicine offers increased clinical precision in the management of malignancies. In order to successfully translate laboratory findings into clinical practice, thorough study of nanomaterial properties is necessary to characterize compound stability, excretion rates, safety profiles and metabolism, as well as identify possible physiological effects of short and long-term exposure. Adoption of diagnostic and treatment gold standards, preclinical and clinical evaluation protocols, as well as large-scale manufacturing specifications for nanomaterial drugs or vectors, is similarly mandated. Finally, although many nanomaterials offer remarkable efficacy with relatively few side effects, much interdisciplinary research remains to be performed for the purposes of optimizing their clinical value and minimizing costs of clinical applications. Fortunately, including the authors of this topic, a multitude of other talented scientists are working as hard as ever to accomplish the aforementioned objectives and improve patient outcomes.

## Author contributions

All authors listed have made a substantial, direct, and intellectual contribution to the work.
